# Quantitative action spectroscopy reveals ARPE19 sensitivity to ultraviolet radiation at 350 nm and 380 nm

**DOI:** 10.1038/s41598-022-17251-7

**Published:** 2022-08-20

**Authors:** Graham Anderson, Andrew McLeod, Pierre Bagnaninchi, Baljean Dhillon

**Affiliations:** 1 Scottish Centre for Regenerative Medicine, University of Edinburgh, 5 Little France Drive, Edinburgh, EH16 4UU Scotland, UK; 2 School of Geosciences, University of Edinburgh, Edinburgh, Scotland, UK; 3 Center for Clinical Brain Sciences, University of Edinburgh, Edinburgh, Scotland, UK; 4 Department of Clinical Ophthalmology, National Health Service Scotland, Edinburgh, Scotland, UK

**Keywords:** Cell death, Mechanisms of disease, Climate and Earth system modelling, Macular degeneration, Lasers, LEDs and light sources

## Abstract

The role of ultraviolet radiation (UVR) exposure in the aetiology of retinal degeneration has been debated for decades with epidemiological evidence failing to find a clear consensus for or against it playing a role. A key reason for this is a lack of foundational research into the response of living retinal tissue to UVR in regard to modern ageing-specific parameters of tissue function. We therefore explored the response of cultured retinal pigmented epithelium (RPE), the loss of which heralds advanced visual decline, to specific wavelengths of UVR across the UV-B and UV-A bands found in natural sunlight. Using a bespoke in vitro UVR exposure apparatus coupled with bandpass filters we exposed the immortalised RPE cell line, ARPE-19, to 10 nm bands of UVR between 290 and 405 nm. Physical cell dynamics were assessed during exposure in cells cultured upon specialist electrode culture plates which allow for continuous, non-invasive electrostatic interrogation of key cell parameters during exposure such as monolayer coverage and tight-junction integrity. UVR exposures were also utilised to quantify wavelength-specific effects using a rapid cell viability assay and a phenotypic profiling assay which was leveraged to simultaneously quantify intracellular reactive oxygen species (ROS), nuclear morphology, mitochondrial stress, epithelial integrity and cell viability as part of a phenotypic profiling approach to quantifying the effects of UVR. Electrical impedance assessment revealed unforeseen detrimental effects of UV-A, beginning at 350 nm, alongside previously demonstrated UV-B impacts. Cell viability analysis also highlighted increased effects at 350 nm as well as 380 nm. Effects at 350 nm were further substantiated by high content image analysis which highlighted increased mitochondrial dysfunction and oxidative stress. We conclude that ARPE-19 cells exhibit a previously uncharacterised sensitivity to UV-A radiation, specifically at 350 nm and somewhat less at 380 nm. If upheld in vivo, such sensitivity will have impacts upon geoepidemiological risk scoring of macular sensitivity.

## Introduction

The question of if, and if so to what degree, solar radiation exposure is involved in the pathogenesis of macular sensitivity has long been the subject of medical contemplation and investigation^1^. Academic interest in the subject widely piqued in the latter half of the twentieth century which ushered in high-altitude flight and atomic ordinance capable of significant retinal injury^2^. However, investigations of the time relied upon a small number of large animals and semi-quantitative means of assessing UVR damage both of which negatively impact upon the statistical power and sensitivity of the observations in describing sub-acute perturbations^3^.

Consequently, contemporary estimates regarding solar photosensitivity of the retina generally favour UVR wavelengths known to be poorly transmitted to the retinal surface^4,5^, thus leading to the conclusion that ‘harmful UVR’ does not reach the retina, despite evidence highlighting paediatric transmission of UV-A^5^. Moreover, the role of lifetime sun exposure in retinal degeneration has been explored by Schick et al*.*^6^ who used questionnaires to determine that sun exposure within the paediatric and occupationally active years of a person’s life are correlated with the loss of visual acuity in later years. However, despite this evidence, contemporary in vitro investigations utilising fully quantitative methods of characterising UVR exposure effects tend to under sample the UV-A band in favour of high-energy visible (HEV; 400–470 nm) and UV-B wavelengths, and in some cases highly biologically effective UV-C wavelengths which do not reach the Earth’s surface within sunlight^7–10^.

Ultimately, this means the quantitative evidence-base regarding solar UVR (280–400 nm) driven effects within the retina, in terms congruent with modern oxidative-stress theories of ageing and disease, remains incomplete. This impedes the accurate geographic modelling of ophthalmologically harmful UVR reaching the Earth’s surface which, in turn, hampers ongoing geo-epidemiological health risk modelling and the shepherding of resources to meet future ophthalmic needs.

In the present study, we sought to characterise the influence of solar UVR upon the cells centrally implicated in the pathogenesis of age-related vision loss, the retinal pigmented epithelium (RPE). Using fully quantitative methods to determine cell viability, oxidative stress burden and tight-junction integrity with a high degree of spectral resolution we sought to create response spectra which can be used in the modelling of ophthalmic risk. Such data may prove valuable when applied to global disease burden modelling within the scope of a changing climate and large-scale demographic processions as well as to infer putative molecules of interest in UVR damage to the RPE.

## Results

### Full spectrum viability assay suggests unique UV-A effects

**Figure 1:**
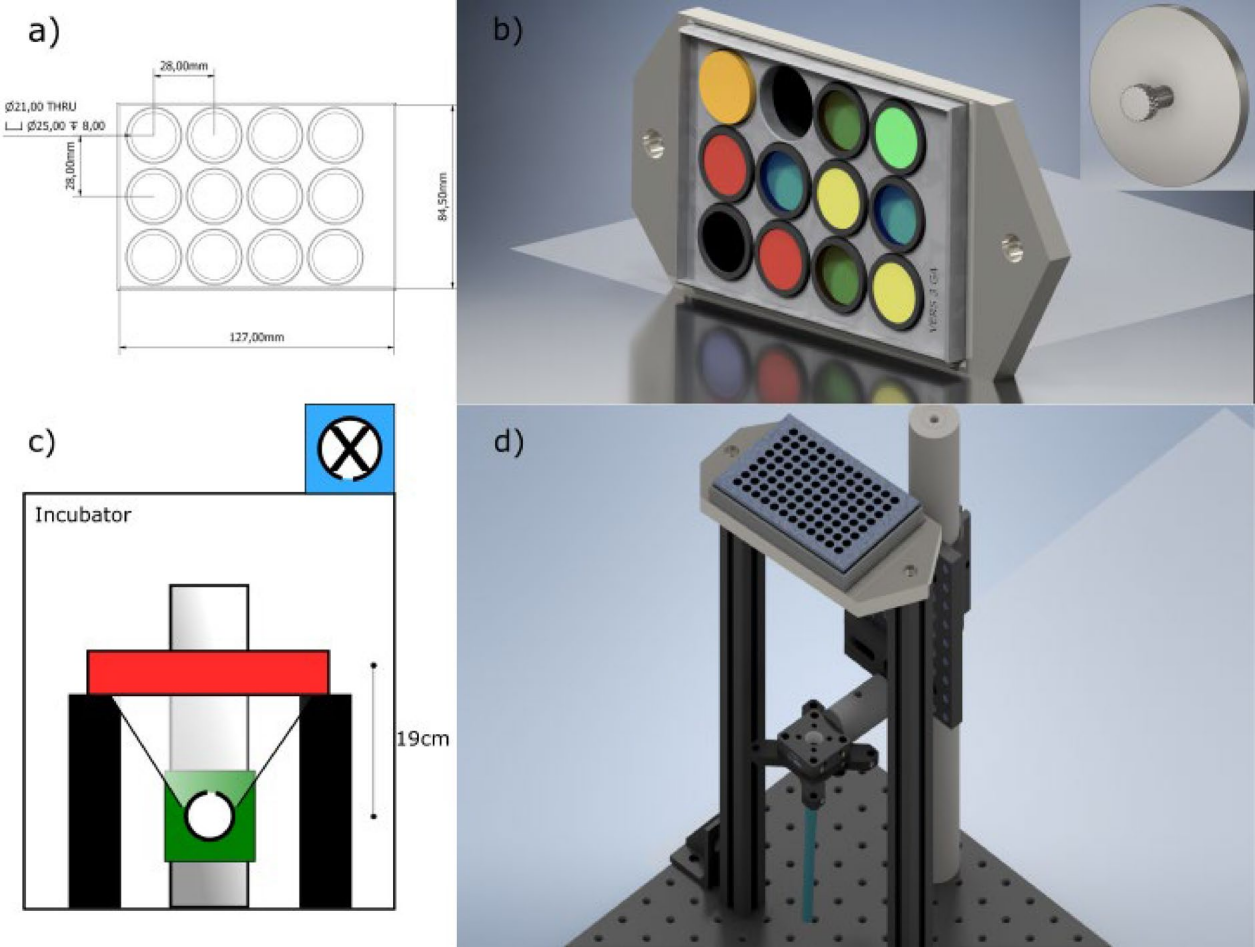
Outline of exposure apparatus. (**a**) Technical drawing of bandpass filter holder used during exposure. (**b**) 3D render of filter holder with filters inserted. Inset 3D render of 'blank' filter, which provided a negative control. (**c**) Schematic of overall design highlighting the position of the culture plate (red box) in relation to the light source (white circle). (**d**) Computer generated mock-up of full optomechanical apparatus with culture plate in place.

**Figure 2:**
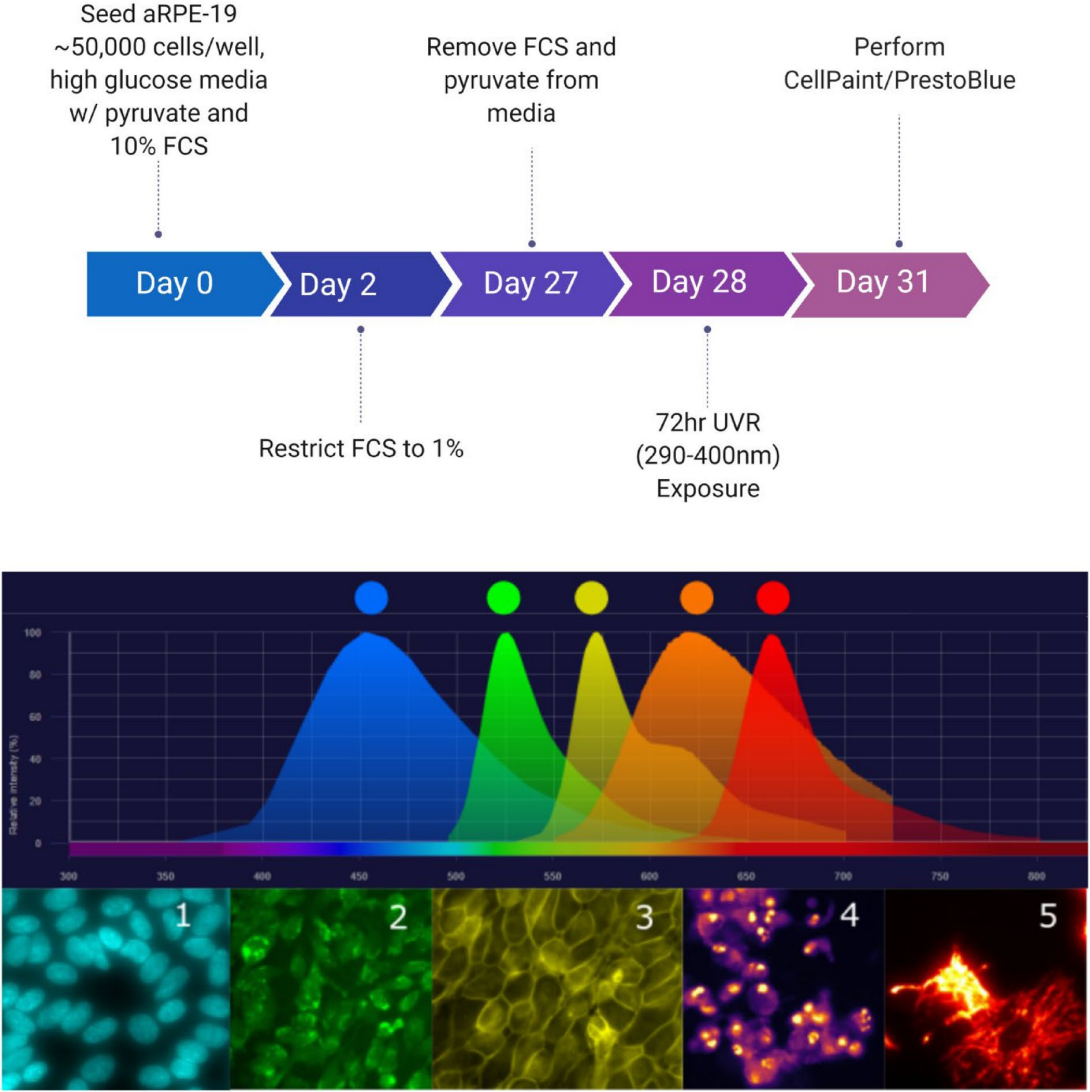
Top—the prototypical workflow of the current project. Middle—the emission spectra of the fluorescent probes used in the current study. Bottom—representative images in ascending order of excitation wavelength. 1 = Hoechst 33,342; 2 = carboxy-H2DCFDA; 3 = CellMask Orange; 4 = Propidium Iodide; 5 = MitoTracker Deep Red FM.

**Figure 3:**
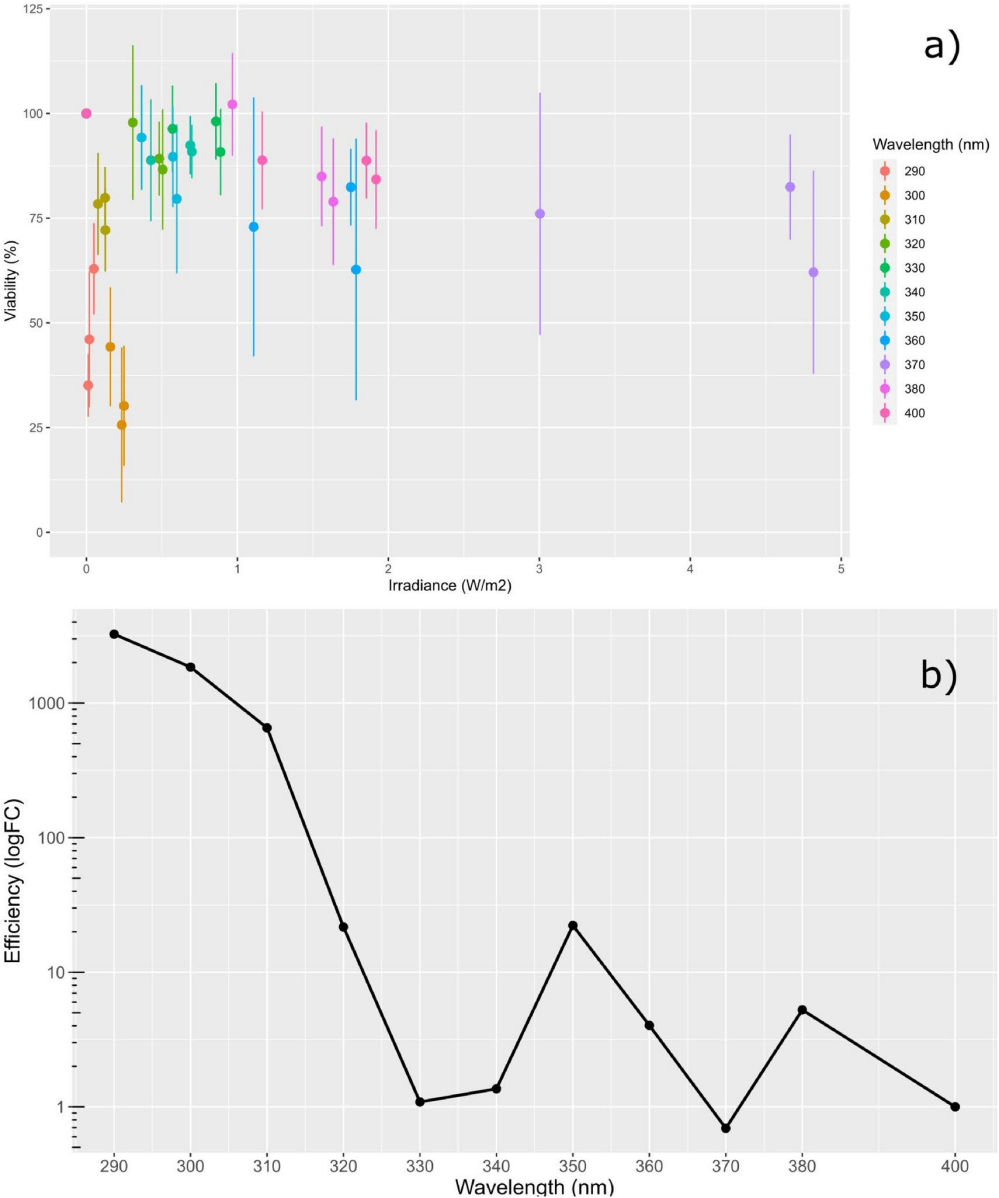
(**a**) Average cell viability, assessed via Prestoblue, as a function of irradiance and wavelength as achieved through the use of neutral density filters, the acute response to UV-B radiation even at low irradiance can be clearly observed. (**b**) The resultant response spectrum based on the slope coefficients of the viability data displaying peaks in phototoxic efficiency at 350 and 380 nm followed by the acute toxicity typical of the UV-B band. N = 3, Error bars = 1 s.d.

Initial investigations coupled a bespoke in vitro exposure apparatus (Fig. 1) to irradiate mature ARPE-19 (see Fig. 2 for culture timeline) and a rapid cell viability agent to quantify wavelength-specific effects. These studies highlighted the clear distinction in photo-damage between the UV-B and UV-A bands (Fig. 3a) with the UV-B wavelengths exhibiting toxic efficiencies several orders of magnitude greater than the UV-A or visible bands (Fig. 3b).

While UV-A radiation was well tolerated by ARPE-19, with a mean decrease of cell viability over the radiation band of 20% of control, response spectra analysis revealed distinct peaks in effects at 350 nm and 380 nm for which a 1.6-fold increase in intensity resulted in a 10% and 30% decrease in cell viability, presenting an approximately 5 and tenfold increase respectively from effects at 405 nm (Fig. 3b).

### ECIS action spectra highlights response to UV-A band

**Figure 4:**
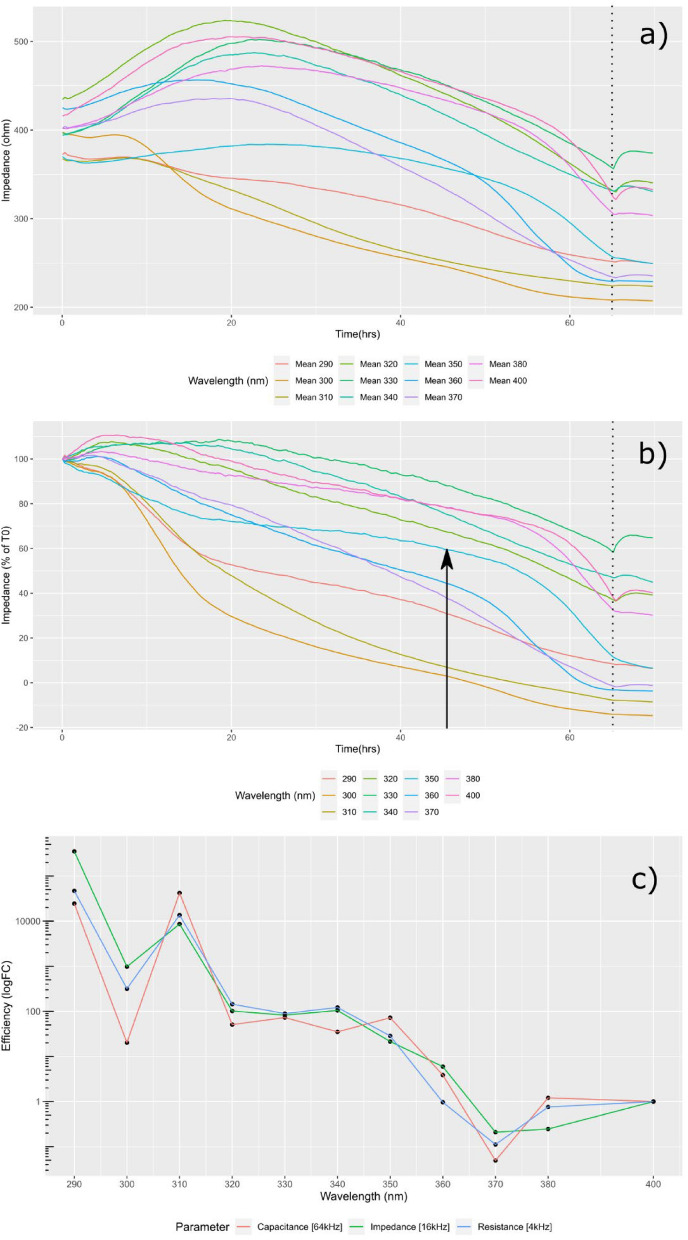
ECIS Data Processing. (**a**) Raw impedance data [16 kHz] averaged for each wavelength. Note the rapid rise in signal at 68 h highlighting the time at which the lamp was doused (vertical dotted line), possibly indicative of the cells re-adhering to the electrode substrate following UVR insult. (**b**) Impedance [16 kHz] data following normalisation to time-zero (T0) and scaling between positive and negative controls. The character of response appeared to vary between UV-B and UV-A wavelengths, where UV-B decline appeared largely monotonic, UV-A response appeared bi-phasic (vertical black arrow), possibly suggesting discrete early and late-stage cellular stress processes. (**c**) Resultant action spectra, normalised to 400 nm, indicating the relative efficiency of each wavelength in reducing each electrostatic parameter to 60% of its initial value. 370–400 nm offered broadly similar weighting in regard to epithelial destabilisation, 320–350 nm appeared to mark a plateau of 100-fold normalised efficiency before giving way to a sustained rise within the UV-B band of more than 1 × 10^4^-fold normalised efficiency. N = 3.

After four weeks in culture, immediately prior to UVR exposure, the media the cells were maintained in was replaced with a custom culture medium free from antioxidants and photosensitisers. Following this, the electrode plate that the cells were cultured upon was docked in the 96-well ECIS station, the lid removed, and the plate exposed to the UVR source continuously for 68–72 h (Fig. 4a).

Within the first 24 h, the majority of wavelengths exhibited a rise in impedance typical of cell responses to changes in ionic balance and CO_2_ following a culture medium change. The exception to this lies in the cells which were irradiated with UV-B radiation, where the inflection in electrostatic response was observed within the first 24 h (Fig. 4b).

Of note when considering the cellular impedance responses to particular wavelengths is the biphasic relationship which emerged over time. This was particularly noticeable in the response to 350 nm UVR which showed a rapid decrease in the first 6 h of exposure followed by an extended plateau until around 45 h where cellular impedance displayed a precipitous decline. This biphasic response suggests two separate events taking place, most likely an initial break down of cellular tight junctions followed by the eventual dissolution of cellular filopodia leading to detachment of the cells from their substrate.

Following normalisation to positive (media-only) and negative (No UVR) controls, action spectra were constructed based on the effective dose required to decrease the electrostatic parameter of interest (impedance [Z], resistance [R] and capacitance [C]) to 60% of their respective time-zero (t_0_) value (Fig. 4c)). The action spectra revealed that longwave UV-A (UV-A_1_) was well tolerated with an apparent trough in toxicity at 370 nm suggesting that, in regard to tight junctional breakdown, it is at least as toxic as high-energy visible (HEV) light. A plateau of toxicity, around 100-fold HEV toxicity, was observed within the shortwave UV-A band (UV-A_2_) beginning at 350 nm and extending to 320 nm followed by a typical rise in toxicity within the UV-B band reaching 10,000-fold of HEV.

### Full depth phenotypic profiling recapitulates 350 nm peak

Initially, variables of interest were chosen according to biological understanding of the processes of UV-B and UV-A pathology. However, following this, an open-ended data-driven approach was employed where all 380 imaging variables were modelled and included in the resultant response spectra.

**Figure 5:**
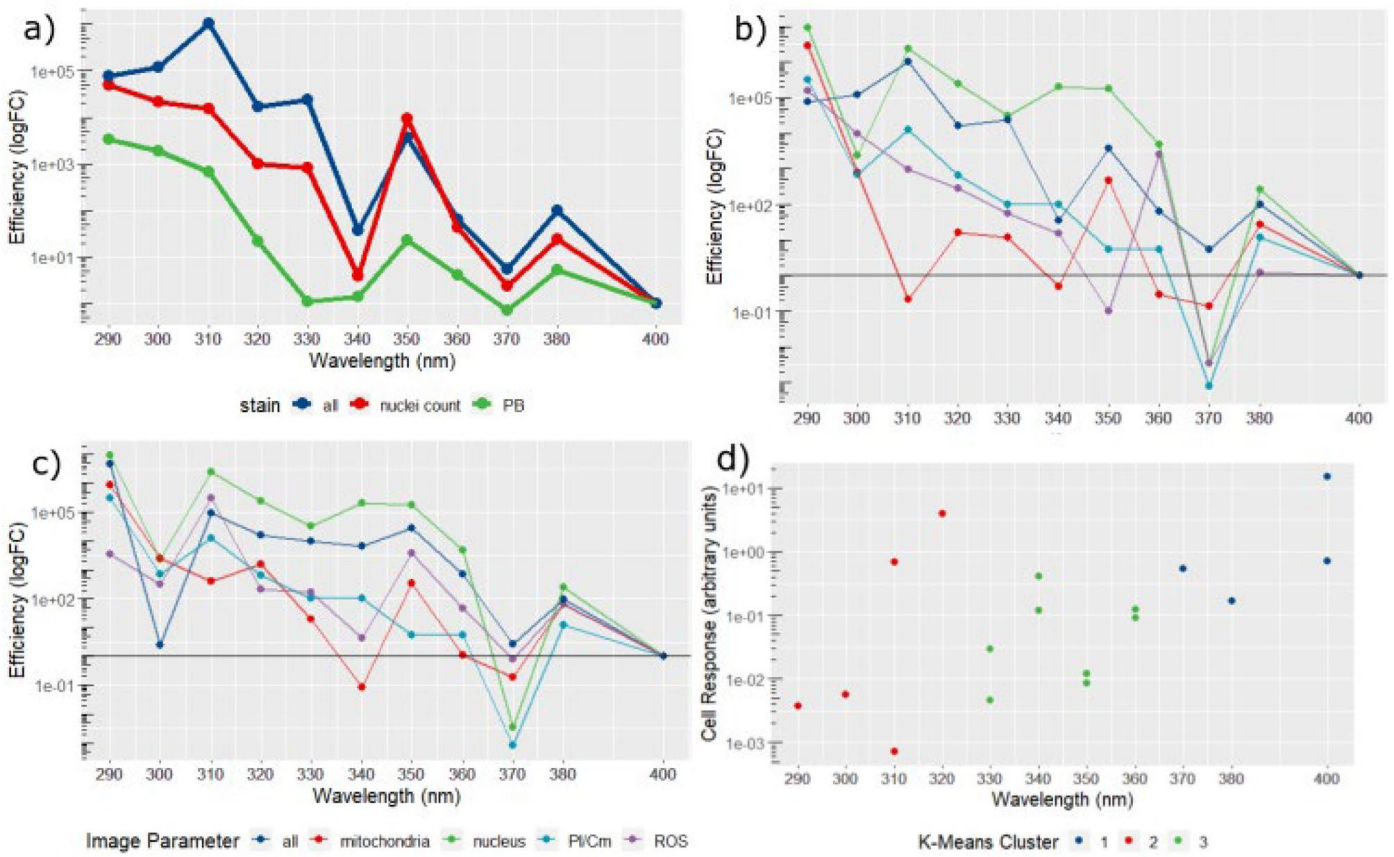
High content image analysis. (**a**) Response spectrum created using all available imaging parameters, recapitulating the 350 nm and 380 nm peaks observed in previous data, presented alongside cell viability and nuclei count response spectra. (**b**) Response spectra from imaging data broken down into major imaging parameters: All, Mitochondria, Nucleus, Propidium Iodide/Cell Mask (PI/Cm), Reactive Oxygen Species (ROS). (**c**) Response spectra created using imaging data produced by the ‘spot’ filtering kernel which successfully recapitulates the 350 nm peak in every compartment except PI/Cm. (**d**) Mapping clusters (k3) onto overall cell response differentiates three main groups, one comprising the UV-B band (290–320 nm) and two within the UV-A band (330–360 nm; 370–400 nm) possibly reflecting discrete mechanisms of action. N = 3 biological replicates.

When considering the overall cell response, that is, the average of all the image parameters for each stain, the resulting response spectra correlate well with the response spectra produced via the PrestoBlue (Invitrogen, MA, USA) and nuclei count assays (Fig. 5a). This suggests that nuclei staining, and the resulting intensity and morphological parameters, provide the most sensitive metric for overall UVR response. Dividing the data by the primary imaging parameters determined by probe: nucleus, mitochondria, reactive oxygen species (ROS), propidium iodide/Cell mask (PI/Cm), it becomes possible to discern which features diverge most from the overall modelled response at each wavelength (Fig. 5b,c).

The use of texture-based image processing techniques, such as the SER filter kernels, make it possible to reliably identify ridge or spot-like cell structures indicative of mitochondrial threads or punctate lysosomes in images with suboptimal signal-to-noise ratios. In the present study, by considering the ‘spot’ texture, it is possible to quantify pockets of ROS activity, condensed nuclear material and condensed mitochondria, all of which are indicative of overt oxidative stress and pre-apoptotic cellular responses. When viewed in this way, the response spectra show a concerted rise at 350 nm and 380 nm in all imaging parameters except the propidium iodide/Cell mask channel suggesting a sub-apoptotic response consistent with chronic oxidative stress (Fig. 5d).

### RPE response falls along distinct UVR bands

In order to elucidate any possible high-dimensional clustering present in the data we undertook Monte-Carlo reference-based consensus clustering (M3C^11^) to provide optimal identification of the number of clusters (represented by the letter *K*) within the data. Consensus clustering assumes that the optimal value for *K* is stable upon resampling, however, statistically robust methods to confirm stability have been lacking^12^. The M3C approach is founded upon the simulation of multiple reference null-data sets (i.e. *K* = 1) based upon the real data supplied by the user and in so doing provides a null-hypothesis for significance testing when comparing higher values for *K*.

Using this method, based upon the coefficient data delivered by the linear models described previously, it was possible to simulate the cumulative distribution function (CDF) and proportion of ambiguous clustering (PAC) scores for the data. Finally, by comparing the real data against the simulated null-data sets, it is possible to generate a p-value regarding the probability of *K* = 1. The M3C analysis suggested that there are 3 or 4 stable clusters within the data, however, upon closer investigation it could be seen that one of the clusters identified referred to data of the blank control suggesting that the true number of clusters was 2 or 3.

Mapping the *K* = 2 clusters to the data simply divided the data into UV-B and UV-A bands. However, mapping clusters based on a *K*-value of 3 subdivided the UV-A response into 330–360 nm and 370–400 nm bands (Fig. 5d). This would indicate that the RPE’s UV-A sensitivity displays a distinct wavelength-dependent response which does not directly map to the established UV-A1 (340–400 nm) and UV-A2 (320–340 nm) bands, suggesting two possibly distinct mechanisms of action for each cluster.

## Discussion

In the present study, we sought to describe the effect of UVR exposure upon the RPE by producing action spectra and response spectra relevant to parameters of interest within retinal ageing and inflammatory pathology, specifically oxidative stress, cell viability and the tight junctional integrity of the RPE. Using the immortalised cell line, ARPE-19, we observed a previously unrealised sensitivity to UVR within the UV-A band, in particular at 350 nm and 380 nm.

The role of UVR in precipitating macular sensitivity remains a subject of some controversy, fuelled in part, by a lack of action spectra regarding the specific means by which UVR would precipitate diseases of ageing such as AMD. Existing action spectra focus entirely on acute phase actions of UVR (i.e. fundus lesions) which are more relevant to occupational light hazards^13,14^ than the chronic exposures expected from sunlight within a public exposure setting^15^. As a result, existing action spectra for the retina and the lens place little to no weight on UV-A effects, suggesting that only UV-B plays a role in UVR damage to these tissues^3,16^.

Recently, Marie and colleagues generated action spectra between 390 and 520 nm for hydrogen peroxide (H_2_O_2_) and superoxide anion (O_2_^·−^) production in A2E-laden porcine RPE^7^. They were able to highlight that A2E acts as a potent photosensitiser capable of increasing H_2_O_2_ production 2–3-fold and doubling O_2_^·−^ content. A2E photosensitisation was highest at 415–455 nm, closely following its absorbance spectrum^17,18^. This was complemented by a concomitant increase in expression of antioxidant genes such as *SOD2* and suppressed respiration rate as quantified via oxygraphy^7^. While A2E does have an absorbance peak within the UV-A band at 335 nm, the researchers did not extend their action spectra beyond 390 nm. However, it follows from the above results that a photosensitive effect would likely be observed if a broader spectrum were constructed.

Subsequently, Marie et al. investigated the spectral sensitivity of the neuronal retina in order to bring depth to our understanding of phototoxicity within retinal degeneration^19^. Through spectroradiometric investigation of the autofluorescent profile of each cell layer of the retina they demonstrated: (1) that the inner-segments of the photoreceptors are the most autofluorescent of the retinal cell layers, (2) that the mitochondria are the primary source of the autofluorescence and (3) that such autofluorescence is highest upon 350 nm excitation. The presence of defined peaks of autofluorescence and phototoxicity at 350 nm strongly imply the presence of a chromophore with maximal absorbance at, or within 10 nm of, 350 nm which resides within the retina. From their observation of peak autofluorescence at 350 nm Marie et al*.* reasoned that the putative chromophores behind the autofluorescence likely belong to the porphyrin or flavin families of photosensitisers^19^. Porphyrins normally exhibit a peak of absorbance around 400 nm, the Soret Band, with subsequent absorbance peaks found at longer wavelengths up to 630 nm, with little absorption within the UV spectrum. However, the molecule bacteriochlorin, a close relation of porphyrin, does exhibit dual absorption peaks at 349 nm and 372 nm^20^.

While exposure to UVR is largely considered a hazard to be avoided, there remain some benefits to UVR providing the dose remains limited. Common examples of UVR’s therapeutic use include its role in vitamin D synthesis and its application as a therapy for infant jaundice. However, evidence of the benefits of UVR within the retina were, until recently, highly limited. Hallam et al. working with patient specific iPS-RPE derived from individuals harbouring a Y402H polymorphism within the *CFH* gene, demonstrated a possible benefit of long-wave UVR exposure to the RPE. In their study, patient-specific iPS-RPE cells and wild type were exposed to 4.5 mW cm^−2^ of 390–410 nm radiation over the course of 5 days. Their results demonstrated a distinct difference in response to UVA/HEV exposure between the genotypes with the mutant line displaying an increase in expression of *SOD2*, *IL*6, *IL18 and IL1β* suggesting a pronounced inflammatory response. Moreover, imaging revealed that the mutant line showed significantly reduced drusen volume relative to the no-UVR control^21^. There is the possibility of epithelial stabilisation following low-grade insult through the multi-nucleation of neighbouring RPE and paracrine activation of the Akt/ERK cell-signalling pathway^22^ but it remains difficult to predict the degree of functional recovery these regenerative instruments would afford a given individual. However, while it is currently unclear which chromophore or pathway is responsible for the photobiomodulatory response described, this finding raises the possibility of using UVA/HEV radiation in a clinical capacity to treat retinal decline within high-risk individuals.

The primary weakness of the present work lies within the reliance upon the spontaneously immortalised cell line ARPE-19^23^. We recognise the limitations of relying upon naïve ARPE-19—that is, RPE free from photosensitisers—and therefore contend that our data represents a conservative estimate of sensitivity. The addition of retinal photosensitisers—such as A2E, all-trans retinal and lipid-rich photoreceptor outer segments, all of which have well characterised UV-A absorption peaks—would only serve to increase the UV-A sensitivity we describe. Conversely, the addition of ectopic anti-oxidants would serve to dampen the toxic effects of UV-A observed. Moreover, to tackle the long-recognised dedifferentiation of ARPE-19 we utilised up-to-date culture methods—which closely mimic induced pluripotent stem cell (iPSC) protocols—capable of ushering ARPE-19 to the fate of mature RPE^24,25^. Based on the work completed so far, it is unclear whether the observed effect is the result of a single photo-oxidative response which differs in degree between 350 and 380 nm or two entirely distinct responses which share a common outcome. To explore this question, one could employ specific anti-oxidants—such as sodium azide or catalase—to inhibit the production of particular oxidative species, or, conversely, utilise super oxide sustaining agents—such as deuterated water—to enhance particular oxidative pathways. With the data produced via these hypothetical means, one might begin to discern the molecular basis of RPE sensitivity to UV-A.

The current study suggests that beyond the UV-B band, the wavelengths 350 nm and 380 nm are the most damaging to the ARPE-19 cell line in regard to cell viability, tight junctional integrity and oxidative stress burden. As such, capturing discrete RPE sensitivity to UV-A vindicates the use of full UV spectral coverage when defining novel UVR effects upon the retina. Future work should focus on determining differential pathways of toxicity between these two wavelengths using mature retinal models and determine how such effects could be altered through supplementation with small molecules capable of modulating lipid handling and oxygen species burden in RPE.

## Materials and methods

### Tissue culture

The immortalised cell line ARPE-19 was purchased from the American Type Culture Collection (ATCC, MA, USA) and, following mycoplasma screening, expanded in T75 culture flasks using Dulbecco’s Modified Eagle Medium supplemented at a 1:1 ratio with Ham’s F/12 solutions (DMEM F/12, Invitrogen, MA, USA) supplemented with 10% foetal calf serum (FCS, Merck, Germany). Once confluent, cells were passaged using porcine trypsin supplemented with ethylenediaminetetraacetic acid (EDTA, Life Technologies, CA, USA). For UVR exposure, amelanistic ARPE-19 cells were seeded into black-walled (µClear, Greiner Bio One Ltd, Germany) and electrode embedded (96w20idf, Applied BioPhysics, NY, USA) 96-well plates at a density of 30,000 cells per well. Cells were maintained at confluence for at least 28 days before UVR exposure in DMEM with 4.5 g L^−1^ glucose, 1 mM sodium pyruvate and 1% FBS to allow for coherent cytoskeletal organisation and epithelial barrier formation^24,25^.

### UVR exposure

A full description of UVR exposure and calculations is given in the “Supplementary information S1”. Briefly, quantification of UVR at 1 nm intervals was carried out using an SR9910-v7 UV–Vis double monochromator spectroradiometer (Irradian Limited, Elphinstone, UK) fitted with a light guide and planar cosine corrected sensor assembly. The culture plate was irradiated inside a dedicated tissue culture incubator (Hera Cell, Heraeus, Germany), kept at 37 °C, 5% CO_2_ and 100% humidity, with an uncollimated beam at 19 cm from the light-guide aperture of the UVR source, a 120 W mercury metal halide epifluorescence lamp (Excelitas Technologies Corp., NY, USA). Exposures took place over 72 h at three irradiance levels, one at full intensity (full irradiance) and two with ø21.3 mm neutral density filters (ND 0.2 and ND 0.4 (ThorLabs Inc., NJ, USA)) mounted within a filter housing of the culture wells (Fig. 1). For each exposure, a negative (dark) control—comprised of a solid disc of black resin—was positioned in one of the available filter positions. Comparison of typical experimental UVR energy doses used in the present study and estimated terrestrial UVR dose confirmed that the UVR doses used in the present study achieved parity with the range of UVR doses experienced by a given person across the life-course (see “Supplementary Data S1”).

### Cell viability

Cell viability analysis was carried out using PrestoBlue (Invitrogen, MA, USA) cell viability reagent according to the manufacturer’s instructions. In brief, culture medium was removed and cells washed with phosphate buffered saline (PBS, Merck, MA, USA) containing calcium and magnesium chloride (referred to hereon as PBS^+/+^). Following washing, one well of the dark control cells was exposed to cell lysis buffer (RIPA Lysis and Extraction Buffer, Thermo Scientific, MA, USA) for 5 min at room temperature to act as a positive control. Next, 120 µL of 1:10 dilution of PrestoBlue (Invitrogen, MA, USA) cell viability reagent and PBS^+/+^ was added to each exposed well. Cells were then incubated at 37 °C and 5% CO_2_ for 20 min to allow the assay to develop. Following incubation, 100 µL of the developed reagent was transferred to a solid white 96-well assay plate and fluorescence was read using a multi-modal plate reader (Ex 520 nm/Em 580 nm; GloMax Explorer, Promega, WI, USA).

### Electric cell-substrate impedance sensing (ECIS)

ARPE-19 cells were seeded onto ECIS culture-ware comprising 96 wells with each well housing 20 interdigitated 300 µm electrodes (96W20Eidf, Applied Biophysics, NJ, USA). Immediately prior to UVR exposure and following tissue maturation, the electrode plate was installed within a 96-well ECIS station. Spectral measurements of electrical impedance (Z), resistance (Ω) and capacitance (C) between 500 Hz and 64 kHz were determined at 11 min intervals during the UVR exposure.

### Cell paint and image analysis

Within 2 h of UVR exposure, the cells were processed for imaging as follows: an equal volume of carboxy-H2DCFDA (Invitrogen, MA, USA) solution diluted at a 1:500 ratio in Hank’s balanced salt solution (HBSS; Sigma-Aldrich, MI, USA) was added to the in situ culture medium and gently mixed by trituration to give a final carboxy-H2DCFDA dilution of 1:1000. The cells were then incubated with the staining solution for 30 min at 37 °C. During incubation, a second staining solution comprising Hoechst 33342 (Sigma-Aldrich, MI, USA) diluted at a ratio of 1:500, CellMask Orange (Invitrogen, MA, USA) diluted at a ratio of 1:500, MitoTracker Deep Red FM diluted at a ratio of 1:500 and propidium iodide (Sigma-Aldrich, MI, USA) diluted at a ratio of 1:1500 were made up in HBSS. During the last 10 min of the 30-min incubation, an equal volume of the second stain solution was added to the first solution in the wells. This was then incubated at 37 °C for a further 10 min. When all staining was complete, 50% washes were performed in triplicate using HBSS.

Live imaging was performed using an automated microscope developed for high-throughput imaging (Operetta, PerkinElmer, MA, USA) the imaging chamber of which was maintained at 37 °C and 5% CO_2_ throughout image capture. Seventeen fields were captured per well at 40X objective magnification (see Fig. 2 for representative images of fluorescent probes).

Image analysis was performed using the Columbus image analysis suite (PerkinElmer, MA, USA). Parameters determined included number of cells, fluorescent intensity, texture analysis (saddle-edge-ridge (SER) textures) and STAR morphology (Symmetry properties, Threshold compactness, Axial properties, Radial properties, and profile) for each cell compartment. Following batch image quantification, all data were exported as a text file for analysis in Excel 2016 (Microsoft Systems, CA, USA) and R-studio^26^.

### Data curation and statistical analysis

PrestoBlue (Invitrogen, MA, USA) data were scaled between the positive (lysed) and negative (no UVR) ‘dark’ controls using the formula: $\left( {\left( {\chi - MIN} \right)/\left( {MAX - MIN} \right)*100} \right)$ where ‘χ’ refers to the individual measurement, ‘MIN’ refers to the lowest value within the dataset and ‘MAX’ refers to the highest value. In a similar fashion, ECIS data were scaled between the positive (no-cell) and negative (no UVR) controls before being normalised to time zero (t_0_). Where possible, impedance, resistance and capacitance data were used to model Rb [tight-junction integrity], α [electrode coverage & cell adhesion] and Cm [membrane capacitance] values (collectively referred to as RbA) as described previously^27,28^.

In order to model the UVR damage efficiency at each wavelength using the PrestoBlue (Invitrogen, MA, USA) data, the processed cell viability for each wavelength and at each irradiance (full irradiance, ND02 and ND04) was used to calculate linear regression coefficients, the slope coefficient of which is indicative of the efficiency of UVR damage. Since the calculated slopes were all negative, they were first squared so they could be plotted logarithmically and normalised to the visible wavelength of 405 nm.

Modelling photodamage efficiency using ECIS was performed by first choosing a ‘common action’ for all wavelengths as a 60% decrease in the electrostatic parameters (Z, Ω, C) from their original values. Through observation of the time-point at which the defined action is achieved, coupled with the knowledge of the light source irradiance, one can calculate the photon dose required to achieve the action. The reciprocal of the dose required to fulfil the action—$\left( {{\text{i}}.{\text{e}}.\;efficiency = \frac{1}{dose}} \right)$—at each wavelength provided an action spectrum for each of the electrostatic parameters. These action spectra were then normalised to 405 nm to provide context in regard to visible radiation effects.

High content imaging data were averaged on a per wavelength basis, then normalised to the no UVR ‘dark’ control prior to action spectrum production. As outlined previously linear regression analysis was performed on the results from the three irradiance conditions (full irradiance, ND02, ND04) for each wavelength and the slope coefficient used to determine efficiency of response. Each exposure was replicated three times for each irradiance condition with four technical replicates present for each wavelength.

The data set was simplified using K-means clustering, supplemented by Monte-Carlo simulation driven consensus modelling, facilitated by the R package M3C^11^, to provide empirical justification for the optimal value of *‘K’.*

All statistical analyses was carried out using R-Studio^26^ and Excel 2016 (Microsoft systems, CA, USA), with data derived from three biological repeats each comprising four technical replicates.

## Supplementary Information

Supplementary Information.

## Data Availability

All data is available from an Open Science Framework repository found at: https://osf.io/fxpwk/?view_only=f6136dfcf9664ffbaefbd683854f2eac.
