# Supplementary material for: Quantitative action spectroscopy reveals ARPE19 sensitivity to ultraviolet radiation at 350 nm and 380 nm
Source: Sci Rep. 2022 Aug 20;12:14223. doi: 10.1038/s41598-022-17251-7 (PMC9392763; doi:10.1038/s41598-022-17251-7)
Supplement: Supplementary file 1 — Supplementary Information. [file 41598_2022_17251_MOESM1_ESM.docx]

# **Supplementary Data**

## Spectral Characterisation

Spectroradiometric quantification of UVR spectra was carried out using an SR9910-v7 UV-VIS spectroradiometer (Irradian Limited, Elphinstone, UK) fitted with a light guide and planar cosine corrected sensor assembly. The sensor was placed 19cm from the uncollimated light-guide aperture of the UVR source, a 120 W mercury metal halide epifluorescence lamp (Excelitas Technologies Corp., New York, USA) within a dedicated tissue culture incubator kept at 37°C, 5% CO_2_ and 100% humidity.

Spectral scans between 260-800 nm, with 1 nm intervals, were performed to establish the source spectrum and the effects of the culture plate media on irradiance (Supplementary figure S1 panel a).

Based on the spectral readings, it was ascertained that at 19 cm from the sensor the light source achieved an irradiance of around 22 W m^-2^ across the UVR region of interest (280-400 nm). In order to measure the influence of the culture plate on tissue irradiance, spectroradiometric readings were taken with the addition of a 96-well black-walled culture plate (#3603, Corning, New York, USA) placed within the illumination apparatus, maintaining a distance of 19 cm between the source and the spectroradiometer sensor, with the lid removed. This provided an irradiance of approximately 7 W m^-2^.

Following the empty culture plate measurements, 100 µL of complete ARPE-19 culture media (DMEM/F12 (Gibco, MA, USA); 1% FCS (Sigma, MI, USA); 1%Pen/Strep (Invitrogen™, MA, USA) was added to each well of the plate and spectroradiometry repeated. This further reduced the irradiance to around 4.5 W m^-2^. Based on these data, the transmission of the media in the 280-400 nm range was calculated to be 60-65% (Supplementary figure S1, panel d)). By subtracting the difference in irradiance between the empty plate, and the plate with media added, from the unimpeded source irradiance it is possible to approximate the irradiance reaching the surface of the cultured cells to be 19.4 W m^-2^.


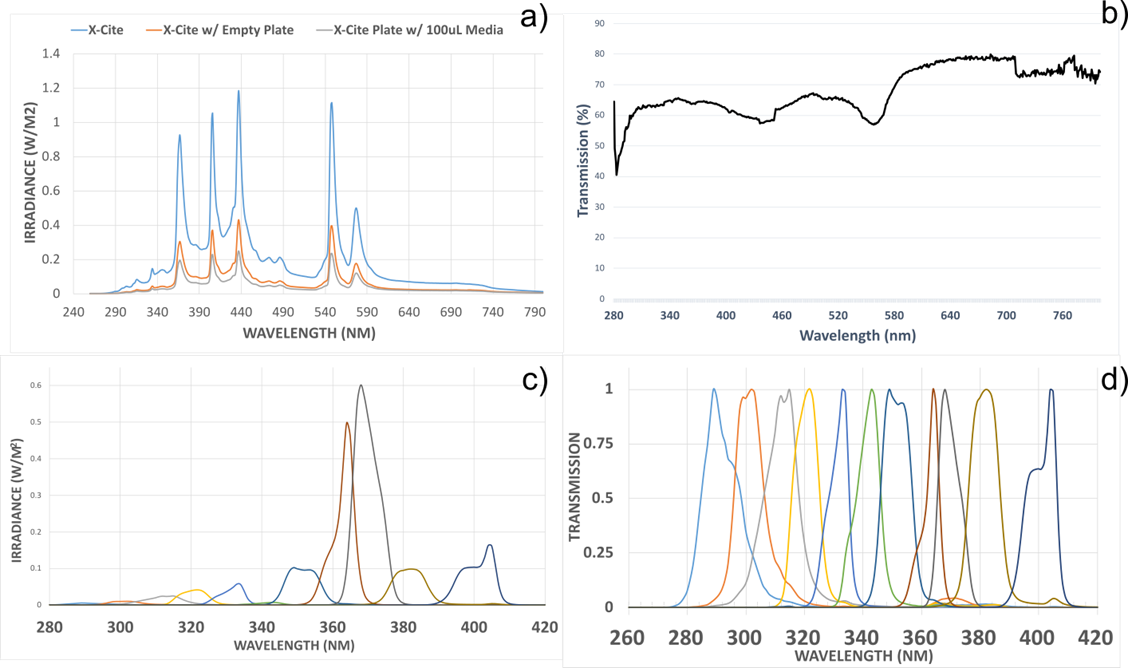


Supplementary Figure 1 -Spectral Characteristics of UVR Exposure Apparatus: a) light source spectrum when uninhibited as well as with cell culture plate and media in place. b) Spectral transmission profile of the media used in the study. c) Unadjusted irradiance data for the bandpass filters used in the study. d) Normalised transmission profiles of the bandpass filters utilised.

## 3D Design & Manufacture of Bandpass Filter Housing

Computer aided design (CAD) software (Inventor 2018, Autodesk Inc., CA, USA) was utilised to design a housing that would allow the Ø21.3mm bandpass filters to be placed under a standard 96-well black-walled culture plate such that cultured cells could be irradiated from below.

The design of the filter holder ensured that each of twelve filter positions covered four wells of a standard 96-well culture plate, thus providing technical replicates for each exposure (Supplementary Figure S2; panel b). Moreover, in order to protect the filters from the humidity within the incubator, two quartz windows were installed on either side of the filter housing within the holder (see Supplementary Figure S1, panel b for quartz window transmission data). To add further protection, the filters were stored within a desiccant container between exposures to extract any moisture caught within their cavities.

## Calculating Tropospheric UVR

The TUV model used in this study *(TROPOSPHERIC ULTRAVIOLET VISIBLE (TUV) MODEL (version 5.0) S. Madronich et al., Atmospheric Chemistry Division National Centre for Atmospheric Research P. O. Box 3000, Boulder, Colorado tuv@acd.ucar.edu)* was downloaded from the NCAR website *[https://www2.acom.ucar.edu/modeling/tropospheric-ultraviolet-and-visible-tuv-radiation-model]* and installed on a computer running Windows 10. All atmospheric inputs were left at their default settings, longitude and latitude were kept at 0° and the model date set to 21/06/2020.

## Calculating UVR Irradiance of the Retina

Based upon published evidence [4,5] we selected a working irradiance of between 0.1-1% of the UVR incident upon the eye reaching the retina. Moreover, based on these findings and the measured transmission properties of the culture media and the black wall plate it is possible to directly compare ambient UVR with the *in vitro* irradiance utilised in the present study.


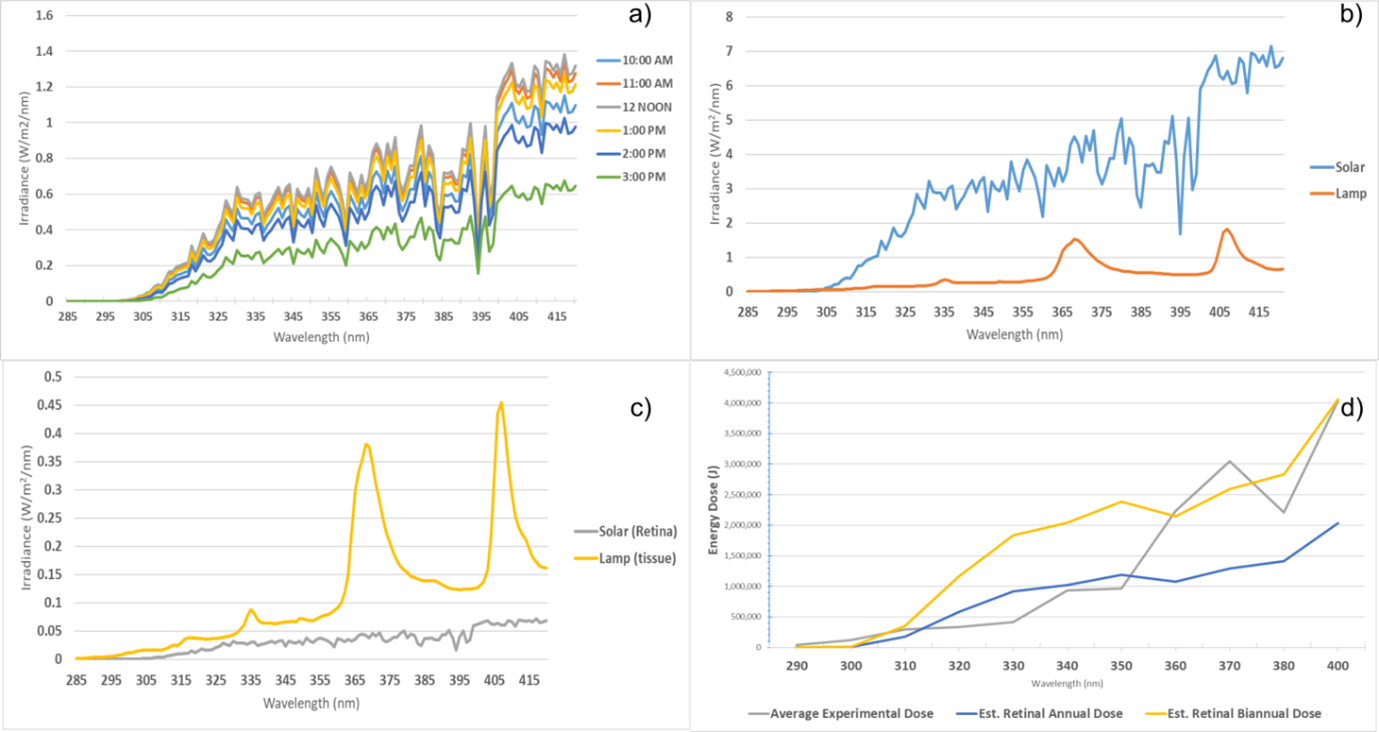


Supplementary Figure 2 -a) Estimated tropospheric ultraviolet radiation irradiance between 280 and 420nm at 0° N, 0° W, on 2020/06/21, between 1000-1500hrs. b) Total daily solar irradiance, from plot a) compared against metal-halide lamp output. c) Solar and lamp irradiances following transmission through the eye and culture plate, respectively. d) Average energy dose (grey line) of in vitro UVR exposures compared against annual (blue line) and biannual retinal dose (yellow dose).

Upon comparison it can be seen that the estimated in vitro tissue irradiance was consistently higher than the estimated solar UVR irradiance of the retina in vivo, the difference is especially large around the mercury emission peaks at 370 and 405nm where the in vitro irradiance is approximately 4.5-fold that of the estimated retinal UVR irradiance (Supplementary Figure S2; panel c). However, the example TUV solar irradiances presented here are instantaneous values at five time-intervals around midday (Supplementary Figure S2; panel a). When considering the uninhibited cumulative dose over two years compared to the dose achieved in exposures used throughout the project the output of the two sources begin to converge (Supplementary Figure S2; plot d).
